# Primary Epiploic Appendagitis

**DOI:** 10.5811/westjem.2015.8.27997

**Published:** 2015-12-01

**Authors:** Po-Jen Yang, Yu-Sung Lee, Chung-Hsun Chuang

**Affiliations:** * Chi-Mei Medical Center, Department of Emergency Medicine, Tainan, Taiwan; † Chang-Gung Memorial Hospital, Department of Emergency Medicine, Taoyuan, Taiwan; ‡ E-Da Hospital, I-Shou University, Department of Emergency Medicine, Kaohsiung, Taiwan

A previously healthy 27-year-old man presented to the emergency department with a three-day history of left lower quadrant pain. He denied fever, nausea, vomiting, or diarrhea. Vital signs were unremarkable, and physical examination revealed tenderness in the left iliac fossa without peritoneal signs. His leukocyte count and C-reactive protein were slightly elevated. On abdominal computed tomography (CT) (Figure), a fatty ovoid mass abutting sigmoid colon demonstrated the infarcted or inflamed appendix epiploica. A surrounding hyperdense rim (hyperattenuating ring sign) represented the inflamed visceral peritoneal covering, and the central linear hyperdensity corresponded to the thrombosed central vessel.^1^ The patient was treated with pain control and intravenous hydration, and was discharged uneventfully five days later.

Epiploic appendages are fat-filled, serosa-covered, pedunculated structures located on the antimesenteric border of the colon. These structures are between 1–2cm thick, 0.5–5cm long, and the size and number increase in the lower abdominal quadrants (57% in the rectosigmoid junction and 26% in the ileocecal region).^1^,^2^ Primary epiploic appendagitis is an acute ischemic inflammation, resulting from torsion of an appendage or spontaneous thrombosis of a central draining vein.^1^,^2^ Patients may experience an abrupt onset of non-migratory, localized pain in the lateral lower quadrants, that worsens with cough and abdominal stretching. Depending on the location of the inflammation, the symptoms may mimic those of acute appendicitis or diverticulitis.^1^ Careful clinical examination and appropriate use of noninvasive imaging studies, including CT or ultrasound, helps in the correct diagnosis preoperatively. Epiploic appendagitis is usually a self-limited illness that can be managed conservatively with pain control.^1^,^2^ Prompt and accurate diagnosis can avoid unnecessary surgical intervention.

## Figures and Tables

**Figure:**
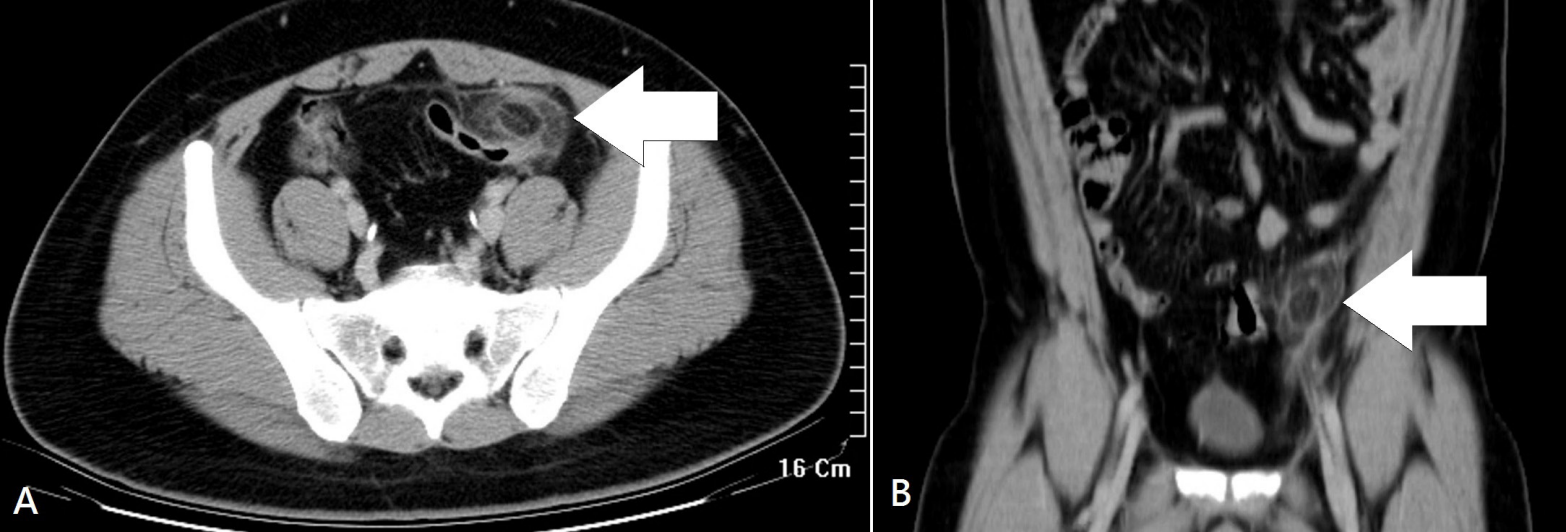
A) Axial and B) coronal abdominal computed tomography showed a pericolonic oval mass (arrow) with a hyperattenuating rim and surrounding fat stranding. A central hyperdense linear lesion corresponded to the thrombosed draining vein.
